# Distribution path robust optimization of electric vehicle with multiple distribution centers

**DOI:** 10.1371/journal.pone.0193789

**Published:** 2018-03-08

**Authors:** Changxi Ma, Wei Hao, Ruichun He, Xiaoyan Jia, Fuquan Pan, Jing Fan, Ruiqi Xiong

**Affiliations:** 1 School of Traffic and Transportation, Lanzhou Jiaotong University, Lanzhou, Gansu, China; 2 School of Traffic and Transportation Engineering, Changsha University of Science and Technology, Changsha, Hunan, China; 3 School of Automobile and Transportation, Qingdao University of Technology, Qingdao Shandong, China; 4 Key Laboratory of Road Traffic Engineering of the Ministry of Education, School of Transportation Engineering, Tongji University, Shanghai, China; Chongqing University, CHINA

## Abstract

To identify electrical vehicle (EV) distribution paths with high robustness, insensitivity to uncertainty factors, and detailed road-by-road schemes, optimization of the distribution path problem of EV with multiple distribution centers and considering the charging facilities is necessary. With the minimum transport time as the goal, a robust optimization model of EV distribution path with adjustable robustness is established based on Bertsimas’ theory of robust discrete optimization. An enhanced three-segment genetic algorithm is also developed to solve the model, such that the optimal distribution scheme initially contains all road-by-road path data using the three-segment mixed coding and decoding method. During genetic manipulation, different interlacing and mutation operations are carried out on different chromosomes, while, during population evolution, the infeasible solution is naturally avoided. A part of the road network of Xifeng District in Qingyang City is taken as an example to test the model and the algorithm in this study, and the concrete transportation paths are utilized in the final distribution scheme. Therefore, more robust EV distribution paths with multiple distribution centers can be obtained using the robust optimization model.

## Introduction

The rapid development of e-commerce has a significant impact on our daily lives and has driven the improvement of the logistics industry in cities. Vehicle path planning is the key link in logistics distribution activities. Identifying a reasonable distribution path can reduce the number of vehicles running, shorten transportation mileage to decrease enterprise cost, promote fast response to demands, and promote quality service to satisfy more customers.

Nowadays, most cities in China are experiencing serious air pollution. In the Copenhagen Conference of Climate Change, the Chinese government declared that China would decrease the ratio between GDP and CO_2_ emission to 40%–45% by 2020. The traffic industry, as a major aspect of greenhouse gas resource, is facing strong pressure to decrease its CO_2_ emission. According to the investigation, 60%–70% CO and 40% NO_x_ come from car exhausts. The Chinese urbanization process has increased the number of private car ownership in urban cities and led to a remarkable rise in logistics distribution. Vehicles are the main transport means of city logistics. The growing logistics distribution implies an increase in the number of vehicles and distribution times. Consequently, both private cars and distribution vehicles can worsen air pollution in urban areas.

Transportation and distribution directly affect the cost of good and city development. Owing to the extension of the logistics transport scale, many problems, such as traffic congestion and explosion pollution, hinder the economy’s development and threaten people’s daily life. Logistics enterprises must take responsibility for converting to green and environment-friendly distribution equipment because of the severe energy and environmental crisis. Electric vehicles (EVs) play an important role in new strategic energy automobile reform, thanks to its advantages, including high efficiency, little noise and explosion, low cost, and multiple energy resources. Consequently, developing EVs and employing them in logistics distribution are of practical significance.

An EV can be driven partially or totally by electricity. However, its limited continual driving mileage makes the EV path different from that of the traditional gasoline car due to battery technology. Occasionally, power in the battery cannot cover the whole path mileage, and the EV has to recharge at a given station halfway to its destination.

Research on the logistics distribution optimization of EV is still in the infancy stage. Artemeier et al. considered the minimized electricity cost as the goal and proposed an optimized model of EV transport route based on the shortest path problem with constraints [1]. Conrad and Figliozz studied rechargeable vehicle route planning and established the mixed integer programming model, which took into account the limited continual driving mileage. No actual charging instrument was provided, but vehicles could be charged at specified customer nodes along the running route [2]. Ren used dynamic traffic network to establish a multi-objective optimization model of a charging station layout and optimal scale based on hard time window. Charging cost and investment of station cost were minimized in the model, which provided basic theoretical support for future EV charging stations [3]. Han introduced operation planning and calculating methods for a pure electric bus charging station. The case study and simulation were used to test the method [4]. Worley et al. established the model that combined the recharging station location with the EV route problem [5]. Sevgi et al. proposed a green vehicle route with a time window for short continual driving mileage of EV and insufficient charging facilities. Two heuristic algorithms were used to acquire the minimal driving distance solution [6]. Liu focused on joint distribution scheduling optimization based on EV technology. Ant colony algorithm was used to solve the model as the continual driving time was limited and the charging time was constrained. Similarly, no actual charging equipment was found, and runnable time was considered the main constraint [7]. Li researched the electric bus scheduling problem with limited running mileage, changeable battery, and fast charging. Two algorithms were designed to solve this model, namely, the branch-and-price algorithm of small-scale search and the heuristic algorithm with column generation and local generation for medium- and large-scale applications respectively [8]. Schneider et al. studied the last-mile application in an electric logistics vehicle by considering constraints of customer time, goods weight, and continual driving. The optimization and scheduling model of the electric logistics vehicle are established to avoid long distribution time and useless route, and suitable recharging stations were chosen when the remaining mileage was not enough to meet the distribution demands [9]. Lu and Chen established including “the shortest total distance and the largest remaining power after arriving destination” multi-objective programming with two objects to research the problem of path optimizing of EV [10]. Xiu et al. presented two-phase mathematic programming model based on power station center and adaptive genetic algorithm to solve the real-time dynamic vehicle routing problem [11]. Anagnostopoulou Afroditi et al. introduced and analyzed the one-to-many vehicle routing and scheduling problem with EV, and emphasized that the optimal or approximate optimal solution for large and medium-sized instance problems can be determined by a large number of calculations [12]. Liu et al. aimed at the problem about EV navigation system multiple charging in a long O-D trip, provided Improved Chrono-SPT (ISC) to derive the optimal decision sequence, to achieve the purpose of finding out an optimal routing and charging policy [13]. He et al. were in order to solve the problem of battery electric vehicles (BEVs) drivers select routes, formulated three mathematical models to describe the resulting network equilibrium flow distributions and then found the optimal path [14]. Huang et al. considered the EV load scheduling problem as the robust shortest path problem and used the Simulation-based Policy Improvement algorithm to solve it [15]. Liu et al. established two mathematical models separately with the equivalent path length and the minimum cost as its object function, and used the traditional ant colony optimization algorithm containing punishment factor to solve the models, to achieve the purpose of selecting the optimal path quickly [16]. Okan et al. pointed out that in order to find the minimum cost path for Plug-in Hybrid EV (PHEVs) in a road network with refueling and charging stations, optimal path can be obtained by using the mixed integer quadratically constrained formulation, the discrete approximation dynamic programming heuristic and the shortest path heuristic [17]. Jane Lin et al. considered the vehicle load effect on battery consumption, produced a general EV Routing Problem (EVRP) that found the optimal routing strategy [18]. Yang et al. presented an EV route selection and charging navigation optimization model, in order to reduce the travel costs of EV users and improving the load level of the distribution system concerned [19]. Martin et al. developed a vehicle shortest routes model, which is the shortest path problem for convertible resources and charging stations. And the solutions of the model can be studied by classifying several types of cycles that may occur in the optimal route [20]. Xie and Jiang proposed a new equilibrium traffic assignment problem with side constraints, and a gradient projection algorithm and a labeling algorithm are adopted to solve the problem [21]. Chiara et al. used second-by-second Global Positioning System (GPS) commute data and traffic micro-simulation data to study the energy consumption effect of route selection on BEVs, also, captured the impact of transient behavior on BEV energy consumption (BEVEC) and recovery while braking in a crowded network based on the microscopic BEVEC model [22]. Gao constructed an EV route model with the goal of minimizing the total distribution cost [23]. Geoke and Schneider adopted the adaptive large neighborhood search algorithm in large-scale applications to solve the route planning problem of mixed traffic platoon with time window; the effects of different objectives and share of EV cost of the obtained results were analyzed as well [24].

Apparently, most of the path optimization research mentioned above focused on certainty conditions but seldom analyzed the EV distribution path with uncertainty conditions, an approach that depended mostly on a hypothesis of a priori knowledge and obeying the probability distribution [25–27]. These kinds of models are too sensitive to uncertain data changes [28]. Moreover, the traditional EV distribution path optimization usually considers the distribution sequence of demand nodes as the final result instead of the road-by-road path scheme. In the current study, the EV distribution robust optimization model with multiple distribution centers considers the minimal changeable uncertainty transport time as the goal, where the charging facilities in the network are considered and genetic algorithm is designed to achieve a more robust optimal path scheme.

This paper is organized as follows. The next section presents the robust optimization model of the EV distribution path. The third section proposes the optimization algorithm. The fourth section discusses the case study. The last section presents the conclusion.

## Robust optimization model of the EV distribution path with multiple distribution centers

An EV distribution path with multiple distribution centers can be defined as a distribution service connecting multiple distribution centers to several client demand nodes after an EV is fully charged at the centers in a given area. Each vehicle can provide distribution service to multiple clients. However, each client can only receive one vehicle service. When the remaining electricity in the vehicle is not enough for the next client, the client must visit the closest charging equipment first prior to moving on to the next destination after charging. The vehicle service is completed when it returns to the original distribution center. Each client can receive service from any EV in any distribution center. The optimal distribution path should be determined to minimize the total distribution time.

### Hypothesis of the model

The following hypotheses are proposed to simplify the problem.

1. The amount of goods stored of distribution centers can meet all client node demands.
2. The amount of goods demanded at any client node is not more than the maximum carrying capacity of the EV responsible for this node.
3. The EV does not lose any electricity during loading and unloading operations at the client nodes.
4. The distribution problem is a pure dispatching goods problem, and these goods are of the same type.
5. Sufficient EVs are available at the distribution centers.
6. In one complete transport mission, an EV can serve multi-client demand nodes, but each node can only receive service from one EV, and each EV can pass through the nodes that have already finished the service numerous times.
7. In one transport mission, the total demand of the EV distributing to client nodes is not more than the permissive carrying capacity of the EV.
8. The EV is fully charged before departing from the distribution center or after visiting the charging facility. Moreover, the charging time is fixed.
9. The ratio of consumed electricity of the EV is standard, and the amount of power consumption is proportional to the distance.
10. The EV runs at a fixed velocity, and the running time is proportional to the running distance.

### Index system

Undirected connecting network *G* = (*N*,*A*) represents the EV transport network with multiple distribution centers, and the property information of all nodes are described by an adjacency matrix.

#### Set definition

*P*_1_ = {*p*|*p* = 1,2,…*n*′}—set of demand nodes of good
*P*_0_ = {*p*′|*p*′ = *n*′ + 1,*n*′ + 2,…*n*′ + *m*}—set of distribution centers of goods
*P*_2_ = {*p*″|*p*″ = *n*′ + 1,*n*′ + 2,…*n*′ + *m* + *h*}—set of charging equipment
*P*_3_ = {*p*|*p* = *n*′ + *m* + *h* + 1,*n*′ + *m* + 2,…*n*}—set of common nodes in the transport network, excluding distribution centers, demand nodes, and charging equipment
*N* = *P*_0_ ∪ *P*_1_ ∪ *P*_2_ ∪ *P*_3_—set of all nodes
*W*—set of paths in the transport network
$V_{p^{'}}=\left\{k|k=1,2,\ldots K\right\}$—set of EVs, *p*′ ∈ *P*_0_

#### Parameter definition

*s*_*ij*_—running distance between nodes *i* and *j*, (*i*,*j*) ∈ *W*
*f*—power consumption ratio of the unit distance of EV
*v*—running velocity of EV
*h*—full charging time of EV
*F*—battery capacity of EV
$F_{ikp`}^{1}$—remaining electricity in battery after EV *k* runs from distribution center *p*′ to node *i*, $i\in W,p^{'}\in P_{0},k\in V_{p^{'}}$
$F_{ikp`}^{2}$—remaining electricity in battery of EV *k* from distribution center *p*′ when it leaves from node *i*, $i\in W,p^{'}\in P_{0},k\in V_{p^{'}}$
*M*—total amount of EV finishing all transport missions of goods
$Q_{k}^{p^{'}}$—maximum carrying capacity of EV *k*, $p^{'}\in P_{0},k\in V_{p^{'}}$
*q*_*i*_—demand amount of client node *p*, *p* ∈ *P*_1_
*t*_*ij*_—nominal value of road link *ij*, (*i*,*j*) ∈ *W*
*δ*_*ij*_—deviation value of road link *ij* according to the nominal one, *δ*_*ij*_ ≥ 0, (*i*,*j*) ∈ *W*
${\tilde{t}}_{ij}$—changeable value of road link *ij*, ${\tilde{t}}_{ij}\in \left[t_{ij},t_{ij}+\delta_{ij}\right]$, (*i*,*j*) ∈ *W*
$load_{ijk}^{p^{'}}$—carrying goods weight of EV *k* from distribution center *p* running on road link *ij*, (*i*,*j*) ∈ *W*, *p*′ ∈ *P*_0_, $k\in V_{p^{'}}$, $load_{ijk}^{p^{'}}\geq 0$

### Definition of other related parameters

$$\begin{array}{l} x_{ijk}^{p^{'}}=\{\begin{array}{cc} 1 & \mathrm{EV}\;\mathrm{of}\;\mathrm{distribution}\;\mathrm{center}\;p^{\prime }\;\mathrm{chooses}\;\mathrm{road}\;\mathrm{link}\;ij\;\mathrm{and}\;\left(i,j\right)\in W,p^{'}\in P_{0},k\in V_{p^{'}}\\ 0 & \mathrm{otherwise} \end{array}\\ y_{ik}^{p^{'}}=\{\begin{array}{cc} 1 & \mathrm{EV}\;\mathrm{of}\;\mathrm{distribution}\;\mathrm{center}\;p^{'}\;\mathrm{services}\;\mathrm{for}\;\mathrm{client}\;\mathrm{nodes}\;i,i\in P_{1},p^{'}\in P_{0},k\in V_{p^{'}}\\ 0 & \mathrm{otherwise} \end{array} \end{array}$$

### Objective function

The interval value used in this study represents the uncertainty road link time of the EV distribution path problem with multiple distribution centers, that is, ${\tilde{t}}_{ij}$, ${\tilde{t}}_{ij}\in \left[t_{ij},t_{ij}+\delta_{ij}\right]$, where the nominal value of road link *ij* is generated from the ratio of road distance *s*_*ij*_ and EV running velocity *v*. Parameters *T*(*T* ∈ [0,|*W*|]) and *E* are also introduced. The former controls the uncertainty conservative factor showing the fluctuation information of *t*_*ij*_, whereas the latter is the set of changing road links due to the uncertainty time. |*E*| is the number of set E. Assigning different values of *T* can help acquire the variable robustness distribution scheme to control the model robustness. When *T* = 0, *t*_*ij*_ is the constant neglecting time deviation, and the model is the most sensitive to uncertainty time. Specifically, the previous optimized result may change when the uncertainty time of a given road link in the distribution network changes. Along with the continual increase of *T*, the model sensitive degree will decrease with the uncertainty time. As a result, the resulting robustness can be improved. The function of *T* = |*E*| means that all the deviations of uncertainty time of all paths are considered. In addition, the model is the most insensitive and the result is the most conservative. According to the robust discrete optimization, robust optimization model of EV distribution path with multiple distribution centers is established as follows:

$$\min T=\sum_{p^{'}\in P_{0}}\sum_{k\in \{V_{p^{'}}|p^{'}\in P_{0}\}}\sum_{\left(i,j\right)\in W}t_{ij}x_{ijk}^{p^{'}}+\underset{\left\{T|T\subseteq W,\left|T\right|=\Gamma \right\}}{\max}\sum_{p^{'}\in P_{0}}\sum_{k\in \{V_{p^{'}}|p^{'}\in P_{0}\}}\sum_{\left(i,j\right)\in W}\delta_{ij}x_{ijk}^{p^{'}}$$ (1)

$$\sum_{i\in P_{1}}y_{ik}^{p^{'}}q_{i}\leq Q_{k}^{p^{'}},\forall p^{'}\in P_{0},\forall k\in V_{p^{'}}$$ (2)

$$\sum_{p^{'}\in P_{0}}\sum_{k\in V_{p^{'}}}y_{ik}^{p^{'}}=1,\forall i\in P_{1}$$ (3)

$$\sum_{\left(i,j\right)\in W}x_{{}_{ijk}}^{p^{'}}-\sum_{\left(i,j\right)\in W}x_{{}_{jik}}^{p^{'}}=0,\forall i\in N,p^{'}\in P_{0},k\in V_{p^{'}}$$ (4)

$$F_{ikp`}^{2}=F,i\in P_{0}/P_{2},\forall p`\in P_{0},k\in V_{p`}$$ (5)

$$F_{ikp`}^{1}=F_{ikp`}^{2},i\in W,\forall p`\in P_{0},k\in V_{p}$$ (6)

$$F_{jkp`}^{1}\leq F_{ikp`}^{2}-f\cdot S_{ij}\cdot x_{ijk}^{p`}+F\cdot (1-x_{ijk}^{p`}),\forall ij\in W,i\neq j,p`\in P_{0},k\in V_{p}$$ (7)

$$F_{ikp`}^{1}\geq 0,i\in W,\forall p`\in P_{0},k\in V_{p}$$ (8)

Where the objective function (1) minimizes the EV distribution time. Carrying capacity constraint (2) means that each EV cannot load more than its maximum carrying capacity. Client demand node constraint (3) shows that any node can only receive service from one EV. Constraint (4) describes the EV running paths, that is, the EV must return to the original distribution center after finishing all node services. Constraints (5) to (8) represent the battery capacity, where in-car battery is the power source of the EV and its capacity is fixed. Constraint (5) indicates that each EV has the maximum power capacity before departing from the distribution center or just after visiting the charging equipment. Constraint (6) presents the remaining power, which is kept the same before and after visiting the client node. Constraint (7) represents that the ratio of power consumption along distribution path link (*i*,*j*) is *f* that is, the power amount at node *j* is equal to it at node *I* minus the consumption amount along path (*i*,*j*). Constraint (8) ensures that the remaining power is nonnegative and the transport is feasible at any time.

The “max” item observed in objective function (1) cannot be solved directly, further equivalent transformation must be carried out. The above function (1) is conversed into (9) by the robust discrete transforming principle [29].

$$H^{IJ}=\min\left\{\sum_{p^{'}\in P_{0}}\sum_{k\in \left\{V_{p^{'}}|p^{'}\in P_{0}\right\}}\sum_{\left\{i=1:\left(i,j\right)\in W\right\}}^{\left\{I:\left(I,j\right)\in W\right\}}\sum_{\left\{j=1:\left(i,j\right)\in W\right\}}^{\left\{J:\left(i,J\right)\in W\right\}}\delta_{ij}x_{ijk}^{p^{'}}+\sum_{p^{'}\in P_{0}}\sum_{k\in \left\{V_{p^{'}}|p^{'}\in P_{0}\right\}}\sum_{\left\{i=1:\left(i,j\right)\in W\right\}}^{\left\{I:\left(I,j\right)\in W\right\}}\sum_{\left\{j=1:\left(i,j\right)\in W\right\}}^{\left\{J:\left(i,J\right)\in W\right\}}\left(\delta_{ij}-\delta_{IJ}\right)x_{ijk}^{p^{'}}\right\}+T\delta_{IJ}$$ (9)

Then, the optimal time objective is shown as $T=\underset{\left(I,J\right)\in W}{\min}H^{IJ}$.

## Optimization algorithm

**Fig 1:**
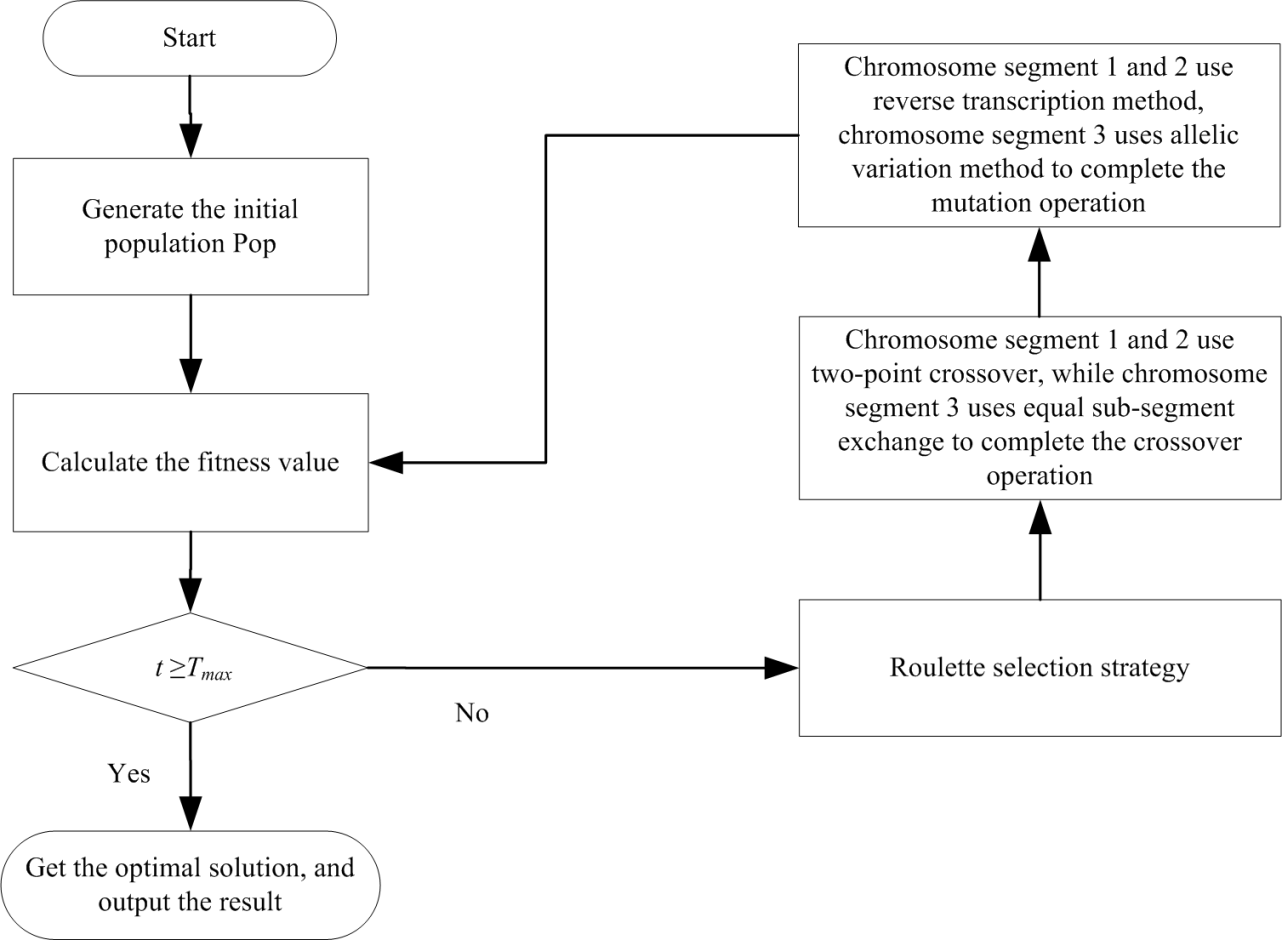
Flow diagram of the algorithm.

The robust optimization model of the EV distribution path is a nonlinear programming model. Therefore, the traditional simple algorithm cannot be used to solve the model effectively. Genetic algorithm can avoid the constraints of problem features, such as linearity, continuity, differentiability, and multimodality. Several feasible results of the optimization problem can be acquired by parallel operation on one chromosome, which is an effective algorithm for large-scale combinational optimization problems [30]. The genetic algorithm is therefore introduced to solve the EV distribution path optimization with multiple distribution centers. The flow diagram of the algorithm is shown in Fig 1, where *T*_*max*_ is the maximum number of iterations.

### Encoding and decoding

The algorithm needs to consider three problems for the EV distribution path problem of multiple distribution centers. The first is choosing the appropriate demand points for each distribution center chooses; next is determining the distribution order of each distribution center; and finally, establishing the path selection of the distribution to demand points and the return to distribution centers. Therefore, the genetic algorithm in this section adopts a hybrid coding method that combines distribution centers, customer demand points, and distribution paths.

**Fig 2:**
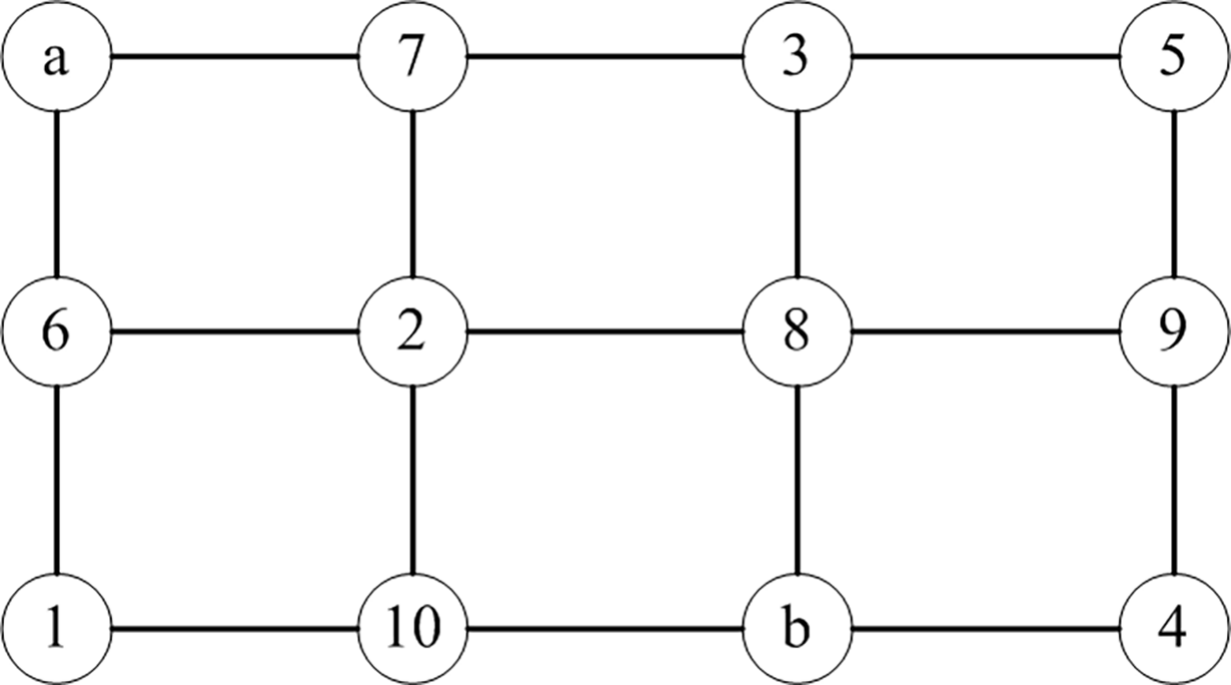
Diagram of the transportation network.

Specifically, a chromosome is divided into three segments. Chromosome segment 1 is the demand points selected from the service by the distribution center. The length of this segment is the quantity of demand points, and its genetic value is the number of each distribution center. Chromosome segment 2 represents the distribution service order of the customer demand points. The length of this segment is the number of customer demand points, and its genetic value is the number of each demand point. Chromosome segment 3 has three components: distribution center–demand point encoding, demand point–demand point encoding, and demand–distribution center encoding. This segment indicates the distribution path, whose length is determined by the number of path nodes (if *s* demand points are serviced by a total of *t* EVs, then the length of chromosome segment 3 is *s+t* and whose genetic value is each node quantity. Chromosome segments 1 and 2 conduct greedy choice according to the loading capacity of EV, and segment 3 conducts neighbor selection between nodes to implement the coding and decoding algorithm of the EV distribution problem with multiple distribution centers. Suppose there is a transport network with 12 nodes (Fig 2). Two distribution centers (nodes a and b) participate in distribution tasks between five demand points (node 1, node 2, node 3, node 4, and node 5). The demands of points are 1.5, 2, 2, 1.5, and 2.5 tons. Vehicle load is 5 tons. A hybrid coding chromosome with three segments is then produced as follows:

Chromosome segment 1: a-b-a-b-a

Chromosome segment 2: 3-1-4-2-5

Chromosome segment 3:

$$\underset{8}{\underset{︸}{7-5-10-a-b-1-8-2-6-3-4-9,\;\overset{12}{\overset{︷}{8-6-b\cdots 5}}\;\cdots \;,\overset{12}{\overset{︷}{9-4-10\cdots 2}}}}$$

From segment 1, demand points 1, 3, and 5 are serviced by distribution center a, while demand points 2 and 4 are serviced by distribution center b. In segments 1 and 2, the distribution order of center a is 3-1-5. Demand points are assigned to appropriate EVs by adopting greedy strategy to meet the conditions of load constraints. Therefore, the distribution order of the first car is a-3-1-a, and the order of the second car is a-5-a. Similarly, distribution center b needs one EV for distribution, and the distribution order is b-4-2-b.

**Fig 3:**
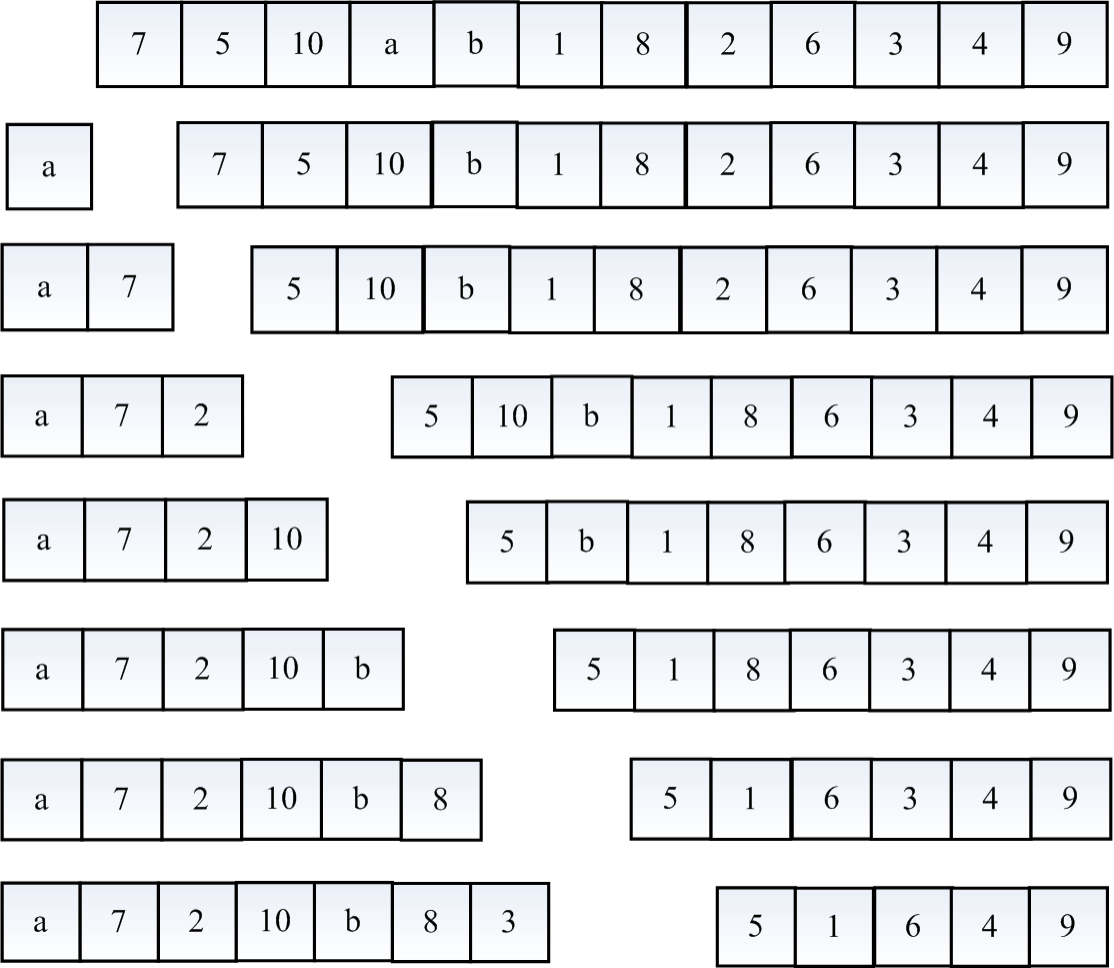
Coding and decoding schematic of chromosome segment 3.

Chromosome segment 3 has eight sub-segments, each of which has 12 genes. These sub-segments are nodes in the network, representing the distribution path between distribution center–demand point, demand point–demand point, and demand point–distribution center. Chromosome segments 1 and 2 show that a center should first provide service to demand point 3. In this study, the coding and decoding processes are described in detail by taking the distribution path between distribution center a and demand point 3 as examples. The coding and decoding schematic of chromosome segment 3 is shown in Fig 3. First, generate an initial sequence of length 12 (7-5-10-a-b-1-8-2-6-3-4-9), traverse the series to find the distribution center a, and find and delete it from the sequence. The length of the sequence is 11. Then, take a as key point, traverse the sequence from the front of the series according to the adjacency relation of the nodes in the transportation network, find the node adjacent to key point a, delete it and take it as key point, and continue to find the next node based on the adjacency relation in the EV transport distribution network until demand point 3 is found. After a node is regarded as a key point, no node in the sequence is found to be adjacent to the key point, and demand point 3 still has not been found, which means that the decoding of the initial sequence cannot acquire the correct distribution path. At this point, the sequence should be re-generated and decoded until the distribution path between two demand points is acquired. Finally, the path length calculates whether the power of the EV arriving at each node is positive and determines whether the path is feasible. Based on the above coding and decoding processes, the distribution path chromosome sub-segment maximum length cannot exceed 12. The encoding and decoding of the other seven sub-segments are the same.

### Fitness function setting

The objective function in the model is always positive. Thus, the individual fitness function can be set as follows:

$$S(x)=\left\{ \begin{array}{l} M-T(x),\;if\;T(x)<M\\ 0,\;if\;T(x)\geq M \end{array}\right.$$ (10)

where *T*(*x*) is the objective function value of the model, and *M* is a given larger number.

### Genetic operator design

The selection operation of the genetic algorithm is performed by roulette selection method. Crossover and mutation operation are designed based on the three-segment encoding style, which can improve the efficiency of the algorithm to search a solution, as well as avoid the generation of a non-feasible solution and premature convergence.

#### Selection operator

This study adopts the roulette wheel selection strategy. In practice, the chromosomes are ordered in accordance with the chromosome target value. Then, the roulette is chunked and calibrated to an adaptive score by a certain distribution according to the size of the population. In the end, a random number between 0 and 1 is generated, depending on which probability region of the random number is present to determine whether each individual is selected. After the selection process, elite law is used to transport the best individual from the previous generation directly into the next generation. Thus, in every process of evolution, the best individual of the next generation must be better than that of the previous generation.

#### Crossover operator

Two-point crossover is used to complete the crossover operation of chromosome segments 1 and 2. First, two individuals are selected as paternal chromosomes by certain probability, arbitrarily selecting two gene loci in the first segment of the selected chromosome as the crossing points. Second, parts between two crossing points of two selected chromosomes are exchanged to form the offspring chromosomes.

**Fig 4:**
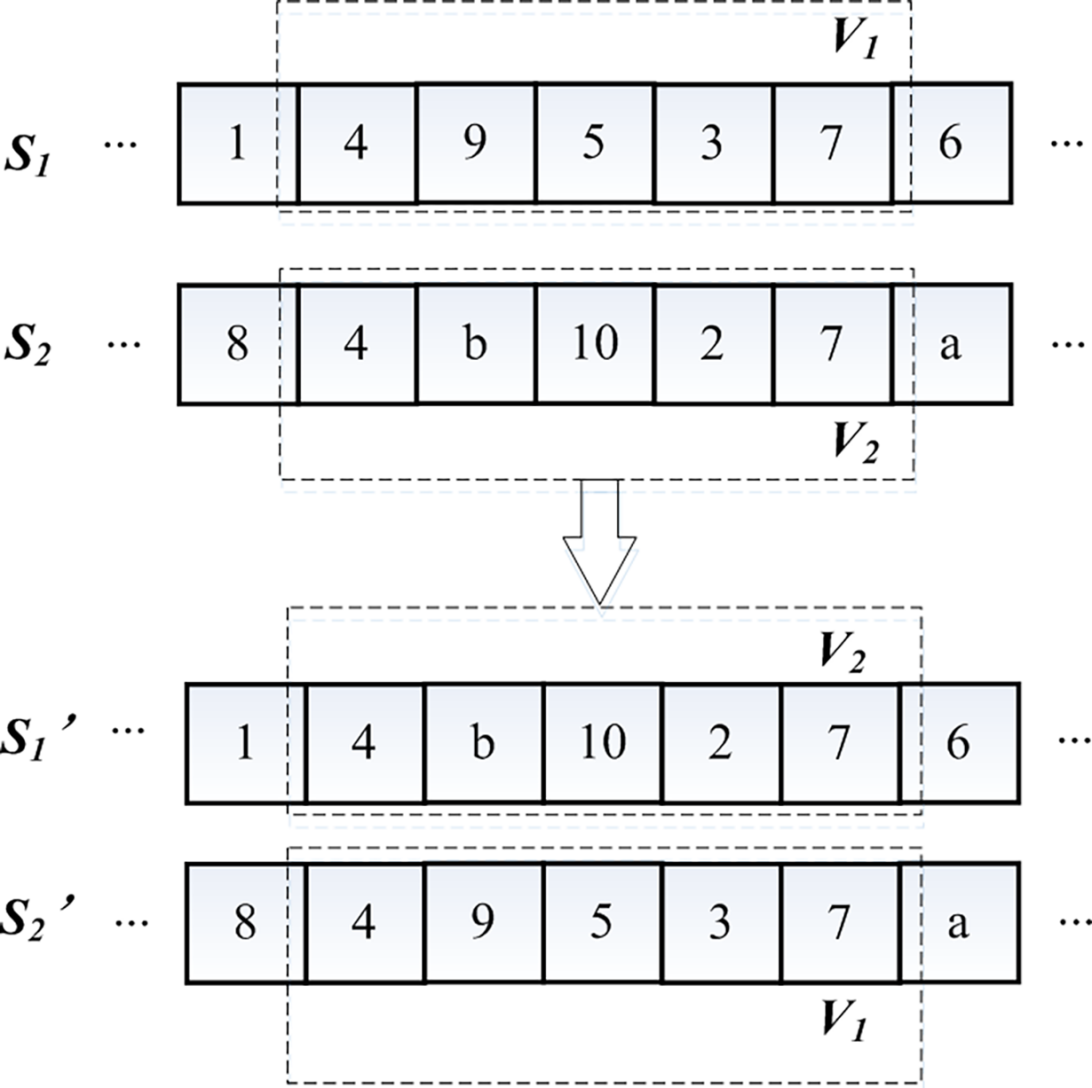
Schematic diagram of equal sub-segment crossover.

With regard to chromosome segment 3, which is transported from distribution center–demand point chromosome sub-segment, demand point–demand point chromosome sub-segment, and demand point–distribution center chromosome sub-segment, in this study, if the first and last nodes of two sub-segments in chromosome segment 3 have the same genetic value, then they are called equal sub-segments. As shown in Fig 4, two chromosomes *S*_*1*_ and *S*_*2*_ with equal sub-segments are selected as the parent. If the crossover probability is satisfied, then equal sub-segments *V*_*1*_ and *V*_*2*_ of two chromosomes will be exchanged to obtain the offspring chromosome. This crossover method is used to ensure the diversity of the offspring chromosomes produced after crossing, ensure its feasibility, and improve the efficiency of the algorithm.

#### Mutation operator

Mutation operation of chromosome segments 1 and 2 is accomplished by the reverse transcription method. Two gene loci are randomly determined in the chromosome segment. If the mutation probability is met, then the reversal operation will be performed on the gene segment between the two gene loci. For example, chromosome segment 4-|3-5-2| is changed to 4-|2-3-5|.

**Fig 5:**
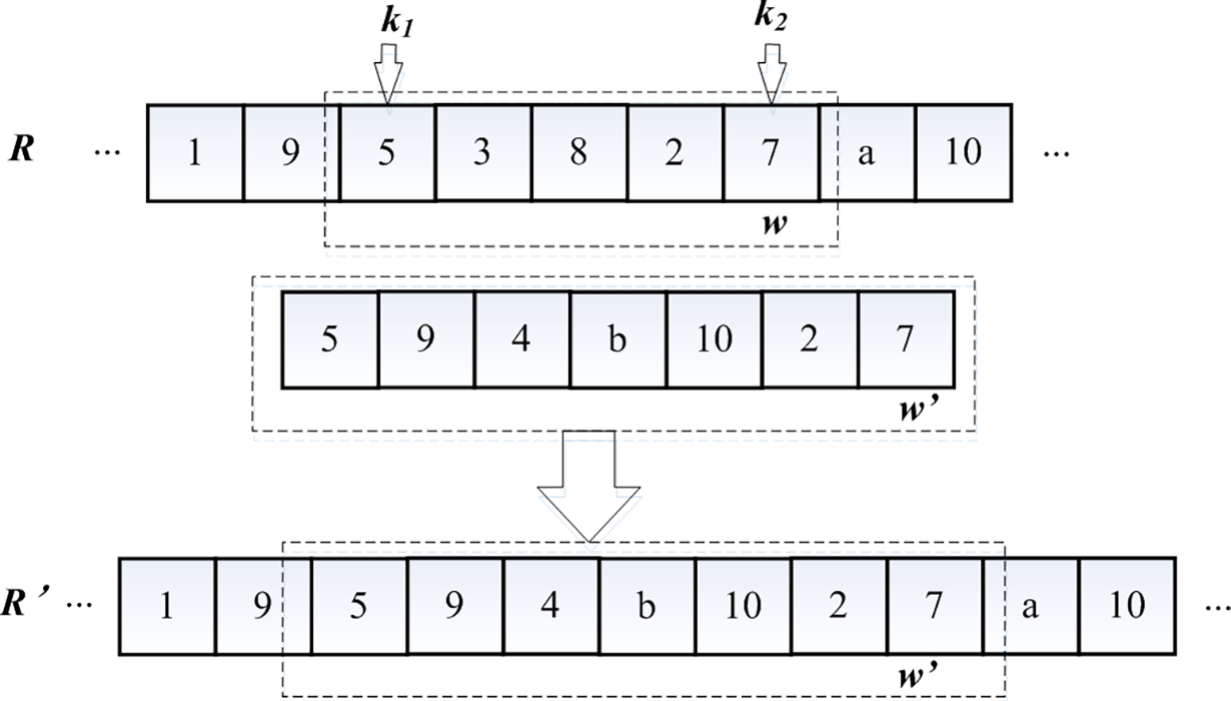
Variation diagram of chromosome segment 3.

Chromosome segment 3 is treated with an allelic variation, because chromosome 3 is the specificity of the path chromosomes generated by the adjacent nodes. In this study, the allele in the chromosome segment is defined as a gene whose start and end nodes have the same genetic value. First, the two nodes *k1* and *k2* on chromosome R are determined to be mutated, and the gene between the two nodes (including *k1* and *k2*) is the mutation gene *w*, as shown in Fig 5. If the mutation probability is satisfied, then the corresponding allele *w*′ will be generated by the coding and decoding methods of chromosome segment 3 to replace the original gene *w* and acquire the new chromosome *R*′. To ensure the follow-up genetic operation, the actual length of the mutated chromosome sub-segment should not exceed the allowed maximum length (the total number of nodes in the EV distribution network is *N*). The use of this mutation method can effectively avoid the generation of infeasible chromosomes.

## Case study

**Fig 6:**
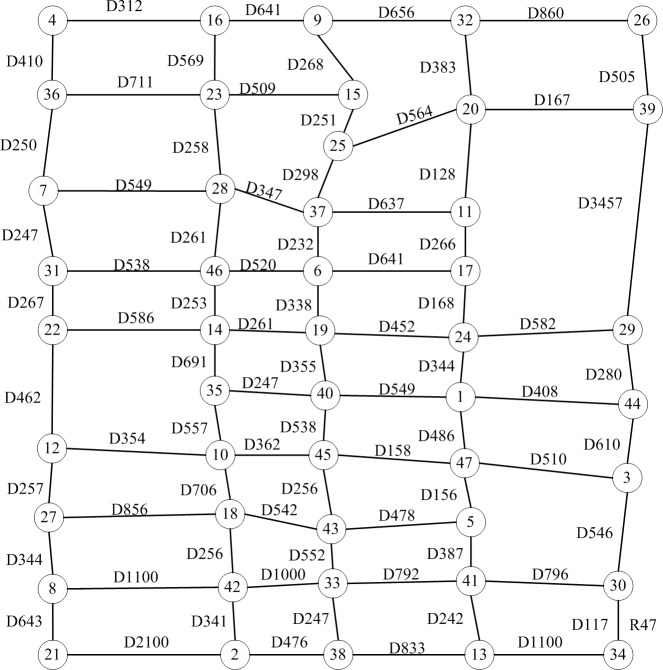
Part of the transport network of Xifeng District in Qingyang, China. Note: Dxxx is the distance of the road.

A part of the road network in Xifeng District of Qingyang City is selected as the sample in the case study. This road network has 47 nodes and 79 sections (|N| = 43, |W| = 79), where nodes 1 to 12 are demand points, nodes 13 to 15 are distribution centers, and nodes 16 to 18 are charging facilities. The distribution center will send EVs to service these 12 demand points. In the case of insufficient power, vehicles will go either to the nearby distribution center or the charging facilities for charging. Demand points are serviced by EVs from distribution centers with capacity tonnage = 5, battery capacity *F* = 800, average speed *v* = 10, and setting power consumption coefficient per unit *f* = 0.2. The demand of each point is shown in Table 1, and the distance of each section *s*_*ij*_ is shown in Fig 6. The nominal value of road travel time *t*_*ij*_ is the ratio of section distance *s*_*ij*_ to average speed *v* of EV, where a certain deviation of the nominal value of the road travel time occurs, assuming that the road travel time deviation *δ*_*ij*_(0 ≤ *δ*_*ij*_ < 0.5*r*_*ij*_) is a random real number.

**Table 1:** Transport task information.

Demand point	Quantity demanded	Demand point	Quantity demanded
1	1.5	7	2
2	1.2	8	1.5
3	0.5	9	1
4	1.7	10	1.2
5	2	11	0.7
6	1	12	0.8

The algorithm is realized in the VC++ 6. 0 platform, where setting population size is 100, maximum evolutionary algebra is 200, reversal rate is 0.1, crossover probability is 0.6, mutative probability is 0.1, and the risk robust control parameters *T* are 0, 20, and 50. The program is run several times to acquire the following results.

**Fig 7:**
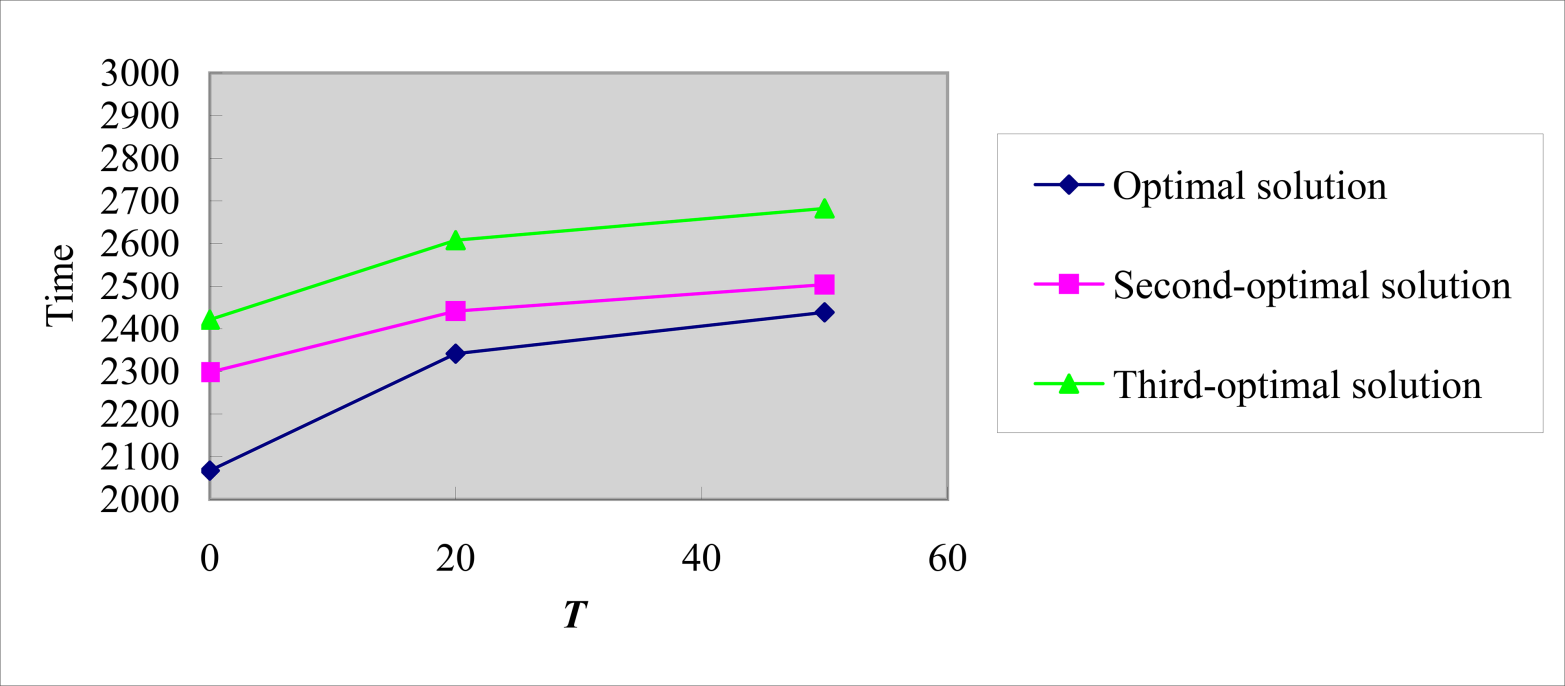
Total distribution time of solution set with different T.

Tables 2–4 show that the client nodes of both distribution centers and each EV servicing are the same in all optimal, second-optimal, and third-optimal solutions with the corresponding *T* value. However, their distribution sequences and paths are not the same at all. The total distribution time of each solution also differs. The EV chooses the nearby distribution centers or charging equipment to finish its mission because numerous client nodes are along its path. Fig 7 shows that the optimal distribution time increases with the increase in robust controlling parameters of path time. Thus, enhancing the solution robustness can, to some extent, cut down the solution optimality. In balancing robustness and optimality, assigning the most suitable value to *T* is a vital issue that depends on the problem features and the judgment of the decision-maker, and thus requires further research.

**Table 2:** Solution set when *T = 0*.

Distribution center	Optimal solution		Second-optimal solution		Third-optimal solution	
	Demand point	Distribution path	Demand point	Distribution path	Demand point	Distribution path
Distribution center 1 (point 13)	(5,1,3)	EV 2: 13-41-5-47-1-44-3-47-5-41-13	(5,1,3)	EV 1: 13-41-5-47-1-47-3-47-5-41-13	(5,1,3)	EV 1: 13-34-30-3-44-1-47-5-41-13
	(2,8)	EV 2: 13-41-33-38-2-42-18-27-8-42-2-38-13	(2,8)	EV 2: 13-38-33-42-18-27-8-42-2-38-13	(2,8)	EV 2: 13-38-2-42-8-42-18-43-33-41-13
Distribution center 2 (point 14)	(12,10,6)	EV 1: 14-22-12-10-35-40-19-6-17-24-19-14	(12,10,6)	EV 1: 14-35-10-12-22-14-19-6-19-14	(12,10,6)	EV 1: 14-22-12-10-35-14-46-6-46-14
Distribution center 3 (point 15)	(9,4,7)	EV 1: 15-9-15-23-16-4-36-7-36-23-15	(9,4,7)	EV 1: 15-9-16-4-36-7-28-23-15	(9,4,7)	EV 1: 15-25-37-28-7-36-4-16-9-15
	(11)	EV 2: 15-25-20-11-37-25-15	(11)	EV 2: 15-25-37-11-37-25-15	(11)	EV 2: 15-25-20-11-20-25-15
Total distribution time	2067.3		2341.6		2438.5	

**Table 3:** Solution set when *T = 20*.

Distribution center	Optimal solution		Second-optimal solution		Third-optimal solution	
	Demand point	Distribution path	Demand point		Demand point	Distribution path
Distribution center 1 (point 13)	(3,5)	EV 1: 13-41-5-47-3-30-34-13	(3,5)	EV 1: 13-41-5-47-3-47-5-41-13	(3,5)	EV 1: 13-34-30-41-5-47-3-47-5-41-13
Distribution center 2 (point 14)	(10,12,8,2)	EV 1: 14-35-10-45-43-18-42-2-42-8-27-12-22-14	(10,12,8,2)	EV 1: 14-35-10-45-43-18-42-2-42-8-27-12-22-14	(10,12,8,2)	EV 1: 14-19-40-35-10-12-27-8-21-2-42-18-10-35-14
	(6,1)	EV 2: 14-19-6-19-40-1-24-19-14	(6,1)	EV 2: 14-46-6-17-24-1-40-19-14	(6,1)	EV 2: 14-46-6-17-24-1-40-19-14
Distribution center 3 (point 15)	(9,4,7)	EV 1: 15-9-16-4-36-7-28-23-15	(9,4,7)	EV 1: 15-25-37-28-7-36-4-16-9-15	(9,4,7)	EV 1: 15-23-28-46-31-7-36-4-36-23-16-9-15
	(11)	EV 2: 15-9-32-20-11-37-25-15	(11)	EV 2: 15-25-20-11-20-25-15	(11)	EV 2: 15-9-32-20-11-37-25-15
Total distribution time	2298.2		2441.7		2503.6	

**Table 4:** Solution set when *T = 50*.

Distribution center	Optimal solution		Second-optimal solution		Third-optimal solution	
	Demand point	Distribution path	Demand point	Distribution path	Demand point	Distribution path
Distribution center 1 (point 13)	(3,5)	EV 1: 13-38-33-43-5-41-30-3-30-41-13	(3,5)	EV 1: 13-34-30-41-5-47-3-47-5-41-13	(3,5)	EV 1: 13-34-30-3-42-5-43-33-38-13
	(2,8)	EV 2: 13-41-33-38-2-42-8-21-2-38-13	(2,8)	EV 2: 13-38-33-42-8-42-2-38-13	(2,8)	EV 2: 13-38-2-42-8-42-33-41-13
Distribution center 2 (point 14)	(12,10,6,1)	EV 1: 14-19-6-17-24-1-47-45-10-12-10-45-40-19-14	(12,10,6,1)	EV 1: 14-35-10-12-10-35-40-19-6-19-40-1-40-19-14	(12,10,6,1)	EV 1: 14-22-12-27-18-10-35-14-19-6-19-40-1-40-19-14
Distribution center 3 (point 15)	(9,4,7)	EV 1: 15-9-15-23-36-4-36-7-36-23-15	(9,4,7)	EV 1: 15-23-28-46-31-7-36-4-36-23-16-9-15	(9,4,7)	EV 1: 15-9-16-23-28-46-31-7-36-4-16-9-15
	(11)	EV 2: 15-9-32-20-11-37-25-15	(11)	EV 2: 15-25-20-11-20-25-15	(11)	EV 2: 15-9-32-20-11-37-25-15
Total distribution time	2421.3		2607.8		2682.4	

In the traditional literature on the vehicle path problem, the algorithm can only work out the client node service sequence. For instance, in this case, distribution center 1(located at node 13) should service client nodes 1, 3, and 5 by the traditional algorithm, and the distribution sequence is 13-5-1-3-13. However, many possible road links are available between the two nodes, such as road links 1-44-3 and 1-47-3 connecting nodes 1 and 3. Moreover, the bigger the network scale is, the more possible path schemes can be chosen. Hence, the distribution sequence is not enough to clarify the concrete transport path. The algorithm in this study can deal with this problem successfully and work out the path with all detailed road links aside from the node sequence. For example, the path scheme of 13-41-5-47-1-44-3-47-5-41-13 is the optimal solution to the client nodes 1, 3, and 5.

## Conclusion

In this study, robust optimization method is used to avoid the dependence that uncertain transport time data are assumed by the probability distribution. The uncertain data are defined in a reasonable interval, which reduces the requirement of the basic data. In the process of solving this problem, a three-segment mixed coding method is designed according to the characteristics of the problem, and the corresponding decoding method and genetic operation are designed according to the coding method, thus avoiding the generation of an infeasible solution in the process of population evolution. On the basis of considering the charging facilities, the robust optimization model of the EV distribution path with multiple distribution centers is established with minimum transport time. The EV transportation and distribution plan is obtained after solving the model and implementing it into the transportation process with the detailed road-by-road path scheme, which has high applicability and economic efficiency. At the same time, setting the different risk coefficients of robust control can obtain an EV distribution plan under different transportation conditions, as well as provide decision support for the choice of EV distribution path with multiple distribution centers.

The use of the geographic information system and construction of a visual optimization platform of an EV distribution path using this method will be the main study objects in the future.
